# Lentiviral gene transfer into the dorsal root ganglion of adult rats

**DOI:** 10.1186/1744-8069-7-63

**Published:** 2011-08-23

**Authors:** Hongwei Yu, Greg Fischer, Guangfu Jia, Jakob Reiser, Frank Park, Quinn H Hogan

**Affiliations:** 1 Department of Anesthesiology, Medical College of Wisconsin, 8100 Watertown Plank Rd, Milwaukee, Wisconsin, 53226, USA; 2 Department of Medicine, Medical College of Wisconsin, 8100 Watertown Plank Rd, Milwaukee, Wisconsin, 53226, USA; 3 U.S. Food and Drug Administration, Center for Biologics Evaluation and Research, Division of Cellular and Gene Therapies, 1401 Rockville Pike Suite 200N/HFM-47 Rockville, Maryland, 20852-1448, USA; 4 Department of Physiology, Medical College of Wisconsin, 8100 Watertown Plank Rd, Milwaukee, Wisconsin, 53226, USA; 5 Milwaukee VA Medical Center, 5000 West National Ave, Milwaukee, Wisconsin, 53226, USA

## Abstract

**Background:**

Lentivector-mediated gene delivery into the dorsal root ganglion (DRG) is a promising method for exploring pain pathophysiology and for genetic treatment of chronic neuropathic pain. In this study, a series of modified lentivector particles with different cellular promoters, envelope glycoproteins, and viral accessory proteins were generated to evaluate the requirements for efficient transduction into neuronal cells *in vitro *and adult rat DRG *in vivo*.

**Results:**

*In vitro*, lentivectors expressing enhanced green fluorescent protein (EGFP) under control of the human elongation factor 1α (EF1α) promoter and pseudotyped with the conventional vesicular stomatitis virus G protein (VSV-G) envelope exhibited the best performance in the transfer of EGFP into an immortalized DRG sensory neuron cell line at low multiplicities of infection (MOIs), and into primary cultured DRG neurons at higher MOIs. *In vivo*, injection of either first or second-generation EF1α-EGFP lentivectors directly into adult rat DRGs led to transduction rates of 19 ± 9% and 20 ± 8% EGFP-positive DRG neurons, respectively, detected at 4 weeks post injection. Transduced cells included a full range of neuronal phenotypes, including myelinated neurons as well as both non-peptidergic and peptidergic nociceptive unmyelinated neurons.

**Conclusion:**

VSV-G pseudotyped lentivectors containing the human elongation factor 1α (EF1α)-EGFP expression cassette demonstrated relatively efficient transduction to sensory neurons following direct injection into the DRG. These results clearly show the potential of lentivectors as a viable system for delivering target genes into DRGs to explore basic mechanisms of neuropathic pain, with the potential for future clinical use in treating chronic pain.

## Background

Chronic neuropathic pain may accompany numerous disease states, but current treatments remain inadequate [1]. There is increasing evidence that the primary sensory neurons and their somata in the dorsal root ganglion (DRG) are critically important sites in the generation of neuropathic pain [2]. Development of pain management therapies to selectively target one or more DRGs would have the clear benefits of directing therapy at the specific involved anatomic pathway while limiting effects on other neuronal populations.

The use of gene transfer vectors based on viruses has emerged as an attractive molecular approach to therapeutically modify the genetic profiles of mammalian cells, including those of the central nervous system [3]. Pre-clinical and clinical studies have begun to investigate the potential use of non-integrating viral vectors, such as herpes simplex virus type 1 (HSV-1) and various serotypes of adeno-associated viral (AAV) vectors, for neuropathic disorders [4-6], but their temporal persistence within neuronal cells following cellular transduction *in vivo *is unproven. Moreover, humoral and innate immune responses may also lead to a diminished biological effect for either of these vector systems [7].

Towards this end, replication-defective lentiviral vectors have been extensively studied for gene therapy applications due to their intrinsic ability to integrate into the host genome, allowing for stable and long-term expression (up to or greater than 6 months) in terminally differentiated neuronal tissue in many mammalian species [8-10]. The need for a long-term therapy is an important feature for any vector that is developed to treat chronic pain. In addition, lentiviral vector integration does not disrupt normal neuronal electrophysiological function [11-13]. Although there are several studies demonstrating successful genetic modification of cultured DRG neurons using lentiviral vectors [14-16] there remains a paucity of data documenting efficient transduction of lentiviral vectors into sensory neurons *in vivo*.

The present study was designed to investigate the vectorological factors that may promote efficient DRG transduction *in vivo*. This is a critical challenge, since it is known that the efficiency of viral vector transduction can be markedly diminished *in vivo *despite optimal performance under *in vitro *conditions [17]. Our experiments examined whether alterations in the lentiviral vector system in terms of envelope coat proteins, viral accessory genes, and internal promoter activity would enhance transduction efficiency and the level of transgene expression in DRG neurons *in vitro *and more importantly, *in vivo*. *In vivo *delivery of vectors to the DRG has been attempted by either direct or remote injection. Specifically, a standard technique exists for intra-DRG injection in human clinical subjects [18], and we have recently devised a direct microinjection technique to reliably and safely deliver high titers of rAAV vector into the targeted DRG region [19]. Alternatively, others have used remote muscle injection for rabies G pseudotyped, but not VSV-G, lentivector deposition [20], although this route preferentially transduced spinal cord motor neurons with limited DRG transduction. In the work reported here, we have compared these approaches. On the basis of our findings, we have concluded that lentiviral vector gene delivery holds great promise for anatomically targeted manipulation of specific neuronal processes that contribute to pain.

## Results

### Optimizing *in vitro *performance of lentivectors

A panel of modified lentivectors was generated by altering several components in our system, specifically: 1) internal cellular promoters, 2) viral accessory proteins, and 3) envelope coat proteins (in a process known as pseudotyping).

#### Constitutively active cellular promoters

As a first step in optimizing lentiviral vector-mediated transduction of DRG neurons, we analyzed various cellular promoters in the context of the lentivector. Because of the inherent variability of primary culture preparations, a cell line was used to initially compare the vectorological performance of the lentivector system. In these initial experiments, we used 50B11 cells, an immortalized rat DRG sensory neuron cell line that mimics the small-diameter subpopulation of DRG neurons *in vivo *by expressing capsaicin-sensitive transient receptor potential vanilloid-1 (TRPV1), and α-calcitonin gene-related peptide (CGRP), binding isolectin IB4, developing currents through TRPV1 channels, generating action potentials, and extending long neurites [21,22]. We have found that these cells expressed TRPV1 and binding IB4 in undifferentiating culture. As reported, neuronal differentiation after forskolin induction were demonstrated in 50B11 cells and β3-tubulin and CGRP were also positive in forskolin-induced differentiated cells (data shown in Additional File 1).

**Figure 1:**
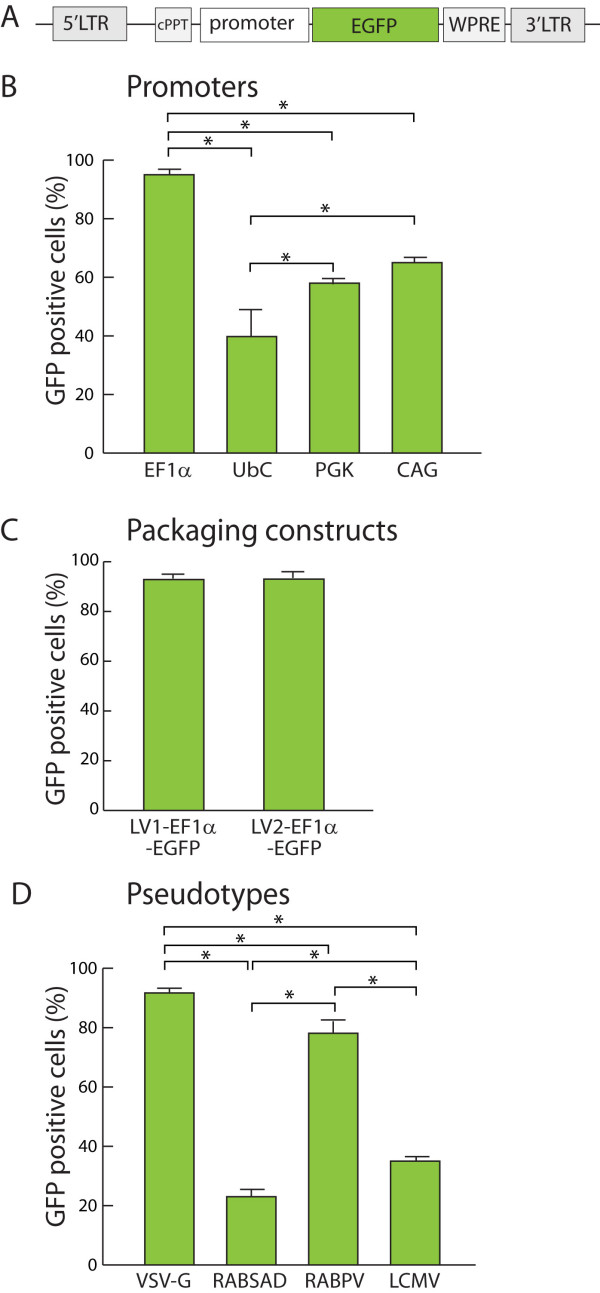
**Lentiviral transduction of immortalized sensory neuronal cells (50B11) *in vitro***. **A**. Schematic representation of the lentivectors. EGFP-expressing lentivector (LV) plasmids carrying a panel of various recombinant promoters were generated. *Cis*-acting sequences included were cPPT (central polypurine tract) to enhance transduction efficiency, and WPRE (woodchuck posttranscriptional regulatory element) to improve transgene expression. **B, C and D**. LV transduction efficiencies in 50B11 cells (MOI = 2) were assessed by FACS analysis. **B**. Transduction efficiencies were compared between vectors with different constitutively active cellular promoters (n = 4 experiments/promoter), including: 1) human elongation factor 1α (EF1α); 2) hybrid promoter containing a human CMV enhancer element 5' to the chicken β-actin promoter (CAG); 3) human ubiquitin C (UbC); and 4) murine phosphoglycerate kinase gene (PGK). **C**. Transduction efficiencies using LVs with (first-generation, LV1-EF1α-EGFP) or without viral accessory proteins (second-generation, LV2-EF1α-EGFP) were compared (n = 8 experiments/packaging system). **D**. Alternative pseudotyping of LVs was performed to determine its effects on transduction efficiency (n = 4 experiments/pseudotype). The envelope coat proteins analyzed were as follows: 1) vesicular stomatitis virus G protein (VSV-G); 2) rabies SAD glycoproteins (RABSAD); 3) glycoprotein from rabies virus PV strain (RABPV); and 4) lymphochoriomeningitis virus envelope (LCMV). *Post hoc *differences between vectors are represented by bars; * p < 0.001.

Lentiviral vectors (Figure 1A) containing enhanced green fluorescent protein (EGFP) as a marker gene were cloned with four different cellular promoters, including the human elongation factor 1α (EF1α) promoter, the composite CAG promoter consisting of the CMV immediate early enhancer and the chicken β-actin promoter, the human ubiquitin C (UbC) promoter, and the murine phosphoglycerate kinase 1 (PGK) promoter. These cellular promoters are believed to be ubiquitously active and have a range of promoter strength [23,24]. Viral promoters were not specifically analyzed in these experiments due to their intrinsic propensity to down-regulate following long-term transduction using various viral and non-viral vector systems [25].

Preliminary experiments applying moderate viral doses (MOI = 5 and 10) onto 50B11 cells resulted in high transduction efficiency and EGFP fluorescent intensity, but with low levels of cell survival, presumably due to the overt toxicity associated with the vectors. In order to improve both cell survival and overall transduction efficiency, the subsequent experiments examining transduction efficiencies were performed using serial transduction steps at low MOI (MOI = 1) on two consecutive days (one application per day), by which toxicity of the lentivectors was minimized. Using this approach, lentivectors containing EF1α-EGFP expression cassette and packaged with the second-generation system produced the most efficient transduction (95 ± 1%; n = 4; P < 0.001) four days after the initial application, compared to other lentivectors containing either the CAG (65 ± 2%; n = 4), PGK (58 ± 2%; n = 4), or UbC (40 ± 10%; n = 4) promoters (Figure 1B). These findings suggest that the EF1α promoter in the context of the lentivector may be the most appropriate for expressing transgenes of interest in DRG neurons.

#### Viral accessory proteins

To determine whether viral accessory proteins that are normally found in wild-type lentiviruses, specifically *vpr, vpu, vif*, and *nef*, can promote increased transduction efficiency in DRG cells, we compared different versions of the lentivector system in which accessory proteins were either present or absent during vector production. EF1α-EGFP vectors complexed with the first-generation packaging system (LV1-EF1α-EGFP), which includes the full complement of viral accessory proteins, produced high transduction efficiency (94 ± 2%, n = 8; Figure 1C). However, vectors generated using the second-generation system (LV2-EF1α-EGFP), which is relatively devoid of the viral accessory proteins found in the first-generation system, produced equally high transduction (93 ± 3%; n = 8). These experiments suggest that viral accessory proteins are not essential for high-efficiency transduction of sensory neurons.

#### Alternate pseudotypes

In the next set of experiments, we swapped the VSV-G envelope with other glycoproteins in a process known as pseudotyping, in order to determine whether changes in the coat protein would alter the transduction efficiency. Lentivectors containing the EF1α-EGFP expression cassette were alternatively pseudotyped with various rhabdoviruses (VSV-G; rabies virus Pasteur vaccine strain, RABPV; and rabies virus SAD strain, RABSAD) or arenavirus (lymphocytic choriomeningitis virus, LCMV) glycoproteins, all packaged using the second-generation system, to determine the transduction efficiency in cultured 50B11 cells. The MOIs for each vector used on the 50B11 cells were adjusted based on functional titers (transducing units, TU) obtained from HeLa cells. As shown in Figure 1D, the VSV-G pseudotyped lentivector was the most efficient in transducing the 50B11 cells (92 ± 2%; n = 4), which was significantly higher (*P *< 0.001) than the other pseudotypes tested in our experiment, including RABPV (78 ± 4%; n = 4), RABSAD (23 ± 2%; n = 4), and LCMV (35 ± 2%; n = 4). EGFP expression images of 50B11 cells after transduction are shown in Additional File 1.

#### Transduction of dissociated DRG cell cultures with lentivectors

To confirm our findings from the 50B11 cell line, the transduction efficiency of lentivectors into DRG neurons was further tested in the primary cultures of DRG cells dissociated from adult rats. Initially, the transduction time course (1-10 days *in vitro*, DIV) and dose response (increasing MOI from 1, 10, 20, 30, and 50) were optimized using VSV-G pseudotyped second-generation lentivectors expressing EGFP driven by the EF1α promoter (LV2-EF1α-EGFP), which exhibited highest transduction rate in 50B11 cells. Unlike the 50B11 cells, primary adult DRG cells were tolerant of the VSV-G pseudotyped lentivector at high MOI (MOI > 20). Overall, the transduction events appeared as a function of time in culture and virus does (data not shown). When cultures were exposed to LV2-EF1α-EGFP at MOI = 1, only an occasional transduction event was observed. At elevated viral doses from MOI 10 to 50, EGFP expression became evident in the cell body of neurons within 48 hr after exposure to LV2-EF1α-EGFP. Increased transduction events were evident as a result of higher MOIs, and EGFP expression appeared maximal after five days *in vitro*, showing positive neurons with axonal projections. In addition, EGFP expression in DRG cultures was also noted in small non-neuronal cells that appeared spindle-shaped with long processes, typical of cultured glia cells, as well as in a few cells typical of fibroblasts [26] . Expression of EGFP remained stable during the lifespan of the cultures, typically more than two weeks.

**Figure 2:**
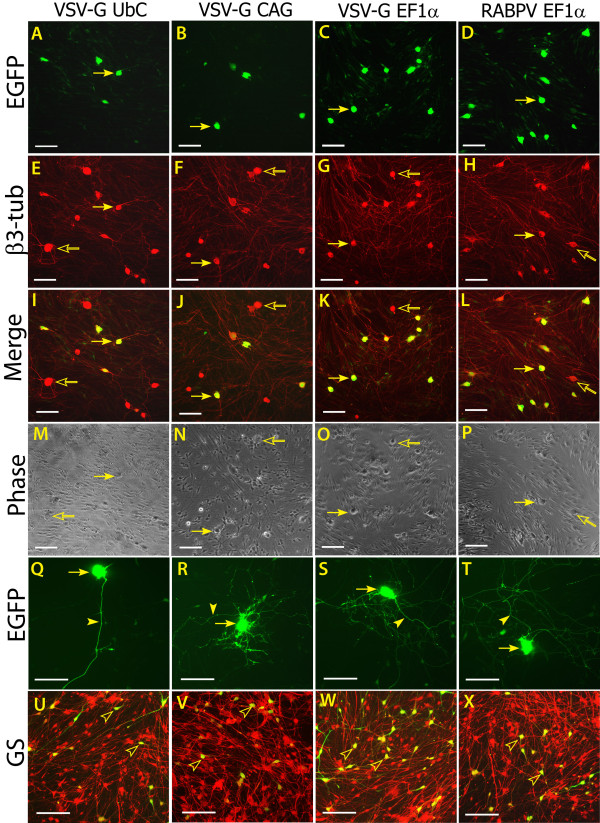
**EGFP expression in dissociated adult DRG cultures after acute exposure to lentivectors**. Primary cultures of DRG cells dissociated from adult rats were transduced at MOI = 20 using VSV-G pseudotyped lentivectors encoding EGFP driven by UbC, CAG, and EF1α promoters, or using RABPV pseudotyped EF1α-EGFP, all packaged by the second-generation system. Cultures were maintained 5-DIV before colocalization immunofluorescence (IF), cell identification, and quantitative analysis. Representative images of the EGFP-expressing dissociated DRG cultures (**A**, **B**, **C**, and **D**) and their correspondent β3-tubulin IF images (**E**, **F**, **G**, and **H**) revealing neuronal patterns, merged data for identification of EGFP-positive neurons (**I**, **J**, **K**, and **L)**, phase-contrast images captured in the same fields (**M**, **N**, **O **and **P)**, and higher magnification images showing neuronal somata and neurite projections in transduced neurons (**Q**, **R**, **S **and **T**), as well as transduced non-neuronal cells colocalized with glutamine synthetase (**U**, **V**, **W **and **X**), are shown in the panels of far-left column for VSV-G UbC, the second-left column for VSV-G CAG, the second-right column for VSV-G EF1α, and the far-right column for RABPV EF1α. Filled arrows point to transduced neurons, empty arrows to non-transduced neurons, filled arrowheads to transduced neuronal projections, and empty arrowheads to transduced glia. Scale bars = 100 μm.

**Figure 3:**
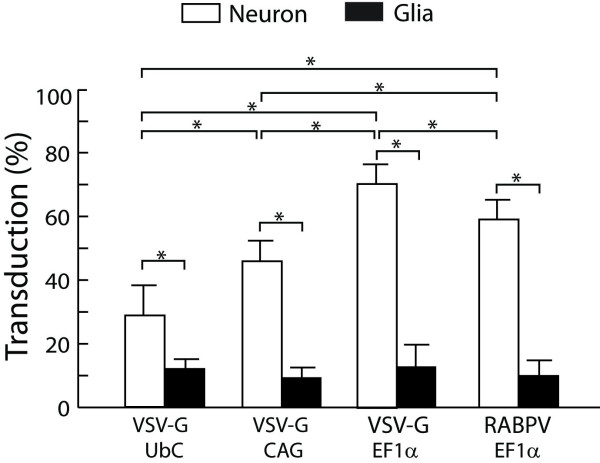
**Quantitative analysis of lentivector transduction efficiency in primary DRG cultures**. Quantification of transduction rates for neurons (identified by β3-tubulin antibody) and glia (identified by glutamine synthetase antibody) in primary dissociated DRG cultures demonstrated vector-specific efficacy for VSV-G pseudotyped vectors containing UbC, CAG, and EF1α promoters and a RABPV pseudotyped vector with the EF1α promoter. *Post hoc *differences between transduction rates are represented by bars; * p < 0.001. (There were no differences in glial transduction rates between the different vectors.)

On the basis of these initial observations, quantitative comparison of *in vitro *vector performance on dissociated DRG neurons was analyzed using a set of OptiPrep-purified and titer-adjusted lentivectors at MOI = 20 for 5-DIV after the initial exposure. Specifically, transduction activities of different cellular promoters were tested, including the EF1α promoter (VSV-G EF1α), the CAG promoter (VSV-G CAG), and the UbC promoter (VSV-G UbC). These promoters had exhibited strong (EF1α), medium (CAG), and weak (UbC) activity in driving EGFP expression in 50B11 cells, respectively. Additionally, the relative transduction efficiency of two pseudotypes, VSV-G and RABPV, were also compared using LV2-EF1α-EGFP, since these showed the best (VSV-G) and the second best (RABPV) performance in transducing 50B11 cells. The results (Figure 2) demonstrated that VSV-G EF1α vector produced the highest neuronal transduction rate (70 ± 7%, n = 4), followed by RABPV EF1α (58 ± 6%, n = 4), VSV-G CAG (48 ± 7%, n = 4), and VSV-G UbC (29 ± 10%, n = 4). Differences between vectors reached significance in all groups (p < 0.001, Figure 3). These results in primary DRG cultures verified a similar variability in transgene expression directed by these promoters as in the test using 50B11 cells (Figure 1). These findings suggest that VSV-G pseudotyped lentivector incorporating human EF1α promoter would be the optimal candidate among the tested vectors for *in vivo *gene transfer to the relatively quiescent cells in the DRG of adult rat.

In addition to neuronal transduction, all these vectors supported robust transgene expression in non-neuronal cells in DRG cultures. Interestingly, although there was no significant difference in glial transduction rates between the various lentivectors (12 ± 2%, 9 ± 2%, 12 ± 7%, and 10 ± 4% for VSV-G UbC, VSV-G CAG, VSV-G EF1α, and RABPV EF1α, respectively), all four tested lentivectors showed significantly lower transduction rates for glia compared to neuronal cells (p < 0.001, Figure 3). This is despite the fact that neurons represent only ~5% of the total cellular population under our culture conditions at 5-DIV.

### *In vivo *assessment of lentivector-mediated gene transfer into DRG in adult rats

Successful transgene expression *in vivo *will be required to take maximal advantage of research and therapeutic applications of vector technology. We therefore investigated factors that might influence *in vivo *DRG transduction, including accessory genes, vector pseudotyping, and site of injection.

#### Influence of accessory genes on in vivo transduction after DRG injection

**Figure 4:**
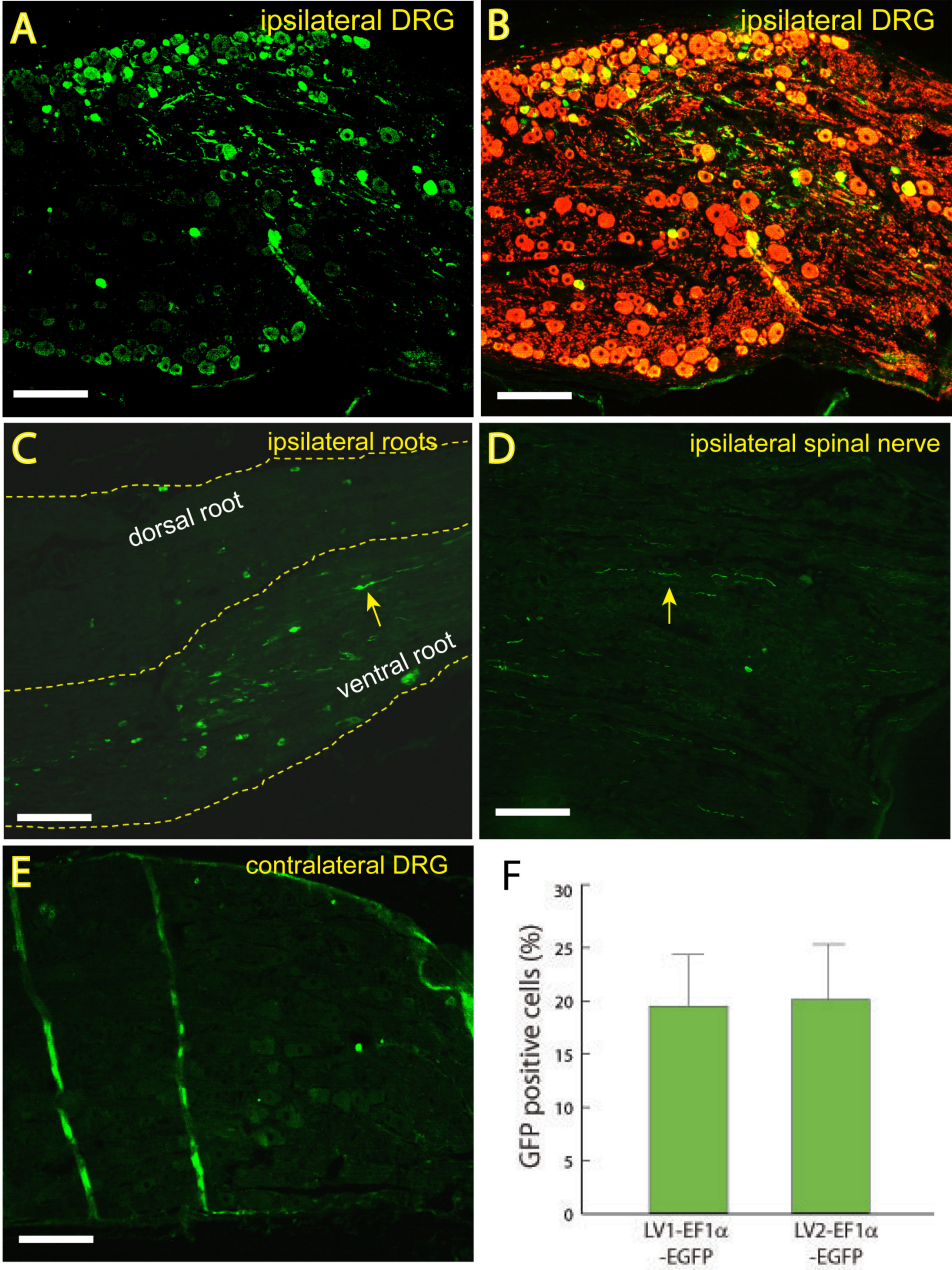
**VSV-G pseudotyped EF1α-EGFP lentivector-mediated gene transfer into dorsal root ganglion in adult rat**. **A, B**. Representative images of immunostained EGFP expression (**A**) and EGFP-expressed neurons co-labeled with β3-tubulin (DRG neuronal marker, **B**) 4 weeks after injection of the LV2-EF1α-EGFP into a L5 DRG. **C, D**. Arrows point to weak EGFP expression in nerve roots proximal to the injection (**C**) and spinal nerve distal to the injection (**D**). No EGFP staining was detected in corresponding spinal cord (not shown) or in contralateral DRGs (**E**; the two vertical streaks are from wrinkles in the section). **F**. Bar graphs represent the percentage of immunostained EGFP positive neuronal cells per total DRG neurons observed in the sections of L5 ipsilateral DRGs injected with VSV-G LV1-EF1α-EGFP (n = 3) and VSV-G LV2-EF1α-EGFP (n = 4). Tissues were collected four weeks after vector injection. Scale bar = 200 μm for A, B, and E; 100 μm for C and D.

**Figure 5:**
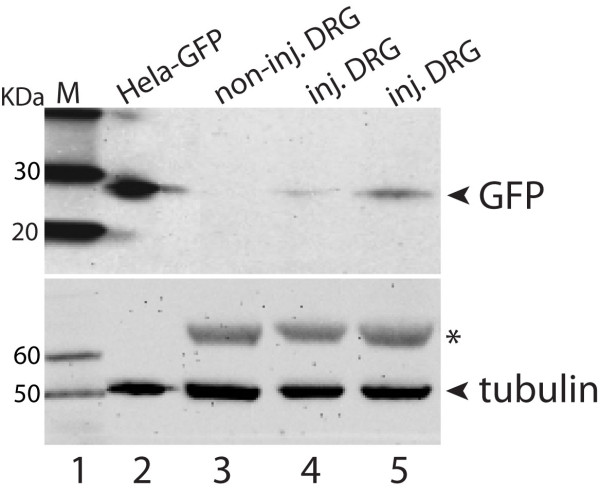
**Immunoblotting analysis of EGFP expression in ipsilateral DRG**. Results in two ipsilateral DRGs (Inj. DRG in lanes 4 and 5) following VSV-G coated LV2 EF1α-EGFP vector, as well as a non-injected DRG (lane 3). Lane 2 is the homogenate from HeLa cells transduced by EF1α-EGFP vector as an EGFP positive control, and lane 1 shows protein standards (MagicMark, Invitrogen). Arrowheads point to the expected size bands for EGFP (top panel), and α-tubulin (bottom panel) as loading control. The additional band in the bottom panel (asterisk) indicates an additional tubulin isoform in DRG.

Because VSV-G pseudotyped EF1α-EGFP was demonstrated to be a good candidate vector by *in vitro *studies, we therefore focused on this vector for our *in vivo *studies of EGFP transgene expression after delivery through direct injection into DRGs. An average total of 1.68 × 10^6 ^TU for purified VSV-G pseudotyped EF1α-EGFP prepared using the second-generation packaging construct (LV2-EF1α-EGFP), or 1.44 × 10^6 ^TU for purified EF1α-EGFP prepared using the first-generation packaging construct (LV1-EF1α-EGFP), were injected in a volume of 2 μl into the fifth lumbar (L5) DRGs. Two out of 24 DRGs harvested at 2-weeks following exposure to the VSV-G pseudotyped LV2-EF1α-EGFP showed only very dim and scattered EGFP signals in sections (data not shown). However, transduction performance assessed four weeks after vector application (n = 10 of 12 ipsilateral DRGs injected by VSV-G pseudotyped LV2-EF1α-EGFP and n = 8 of 10 ipsilateral DRGs injected by VSV-G pseudotyped LV1-EF1α-EGFP) revealed EGFP fluorescence of neuronal somata and axons. Representative images (Figure 4A), photographed from a section of DRG injected by VSV-G pseudotyped LV2-EF1α-EGFP, shows immunostained EGFP expression, which are colocalized with β3-tubulin (Figure 4B), whereas EGFP signal is absent in contralateral DRGs (n = 6, Figure 4E), demonstrating that VSV-G pseudotyped EF1α-EGFP is capable of transducing DRG cells *in vivo *with anatomic selectivity. Fluorescence was also evident in axons both among somata and also in axon fascicles. In ~50% of the injections, weak EGFP signals were detected in nerve roots, variably both ventral and dorsal, proximal to the injection (Figure 4C), and in the spinal nerve distal to the injection (Figure 4D). Identification of EGFP by immunohistochemistry did not reveal any signals in the corresponding spinal cord segment (n = 4, data not shown), indicating anatomic selectivity of transduction with lentivector administered directly to an individual DRG. The transduction levels appeared to be independent of the generation of the lentivector packaging system. Specifically, using either the first- or second-generation packaging system resulted in 19 ± 9% in the VSV-G pseudotyped LV1-EF1α-EGFP and 20 ± 8% in the VSV-G pseudotyped LV2-EF1α-EGFP (*P *> 0.05) of EGFP expressing profiles per total neurons within the field, respectively (Figure 4F). As confirmation of the IHC data, EGFP expression in DRG homogenates was also detected by immunoblots (Figure 5). There was no structural change or infiltrate evident in the DRG apparent by hematoxylin/eosin staining (n = 3 DRGs, data not shown).

#### Transduction of identified sensory neuron subpopulations after DRG injection

**Figure 6:**
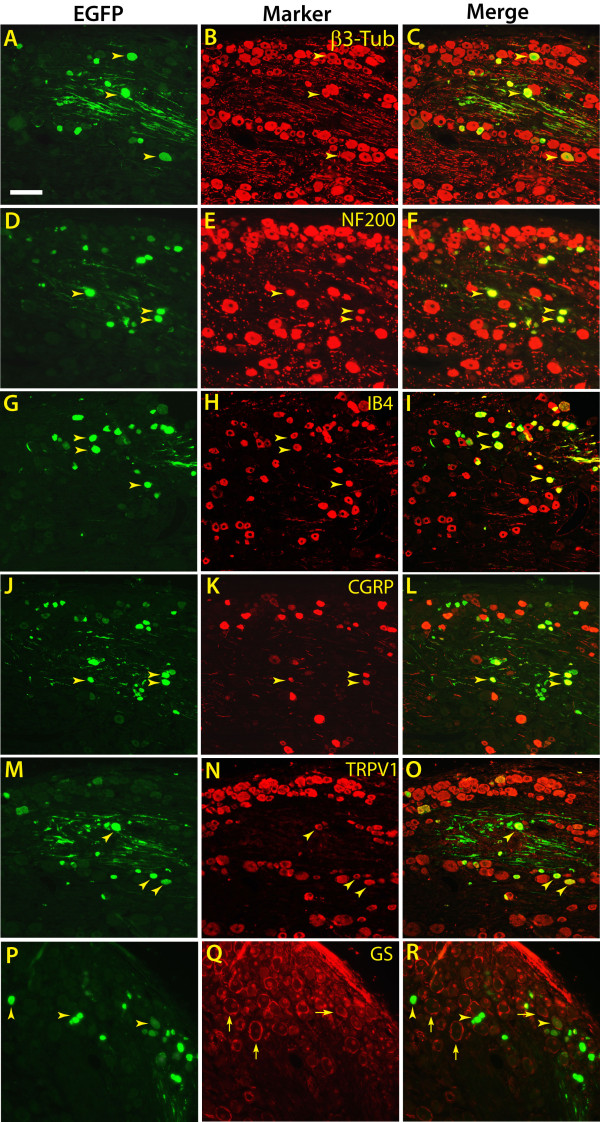
**EGFP expression in subpopulations of primary sensory neurons following VSV-G pseudotyped EF1α-EGFP vector injection into DRG**. 4 weeks after VSV-G LV2-EF1α-EGFP vector injection ipsilateral DRG sections were immunostained with EGFP antibody (green, **A, D, G, J, M, P**) to detect EGFP expressing cells. Neurons were identified by staining for β3-tubulin (β3-Tub, red, **B**). Neurofilament 200 (NF200, red, **E**) labeled myelinated neurons, isolectin IB4 (IB4, red, **H**) labeled non-peptidergic small neurons, α-calcitonin gene-related peptide (CGRP, red, **K**) labeled peptidergic small neurons, and transient receptor potential vanilloid 1 (TRPV1, red, **N**) labeled nociceptive neurons. Satellite glia were labeled with glutamine synthetase (GS, red, **Q**). Arrowheads are examples of EGFP-immunoreactive cells double labeled for a given neuronal marker, evident in the overlays (yellow, **C, F, I, L, O**). In **P**, **Q**, and **R**, the arrowheads denote EGFP-immunoreactive neurons negative for GS, while the arrows indicates rings of satellite glial cells. Scale bar: 100 μm.

Immunofluorescent double labeling was next performed to characterize sensory neuron subpopulations expressing EGFP in DRGs. L5 ipsilateral DRG sections from VSV-G pseudotyped LV2-EF1α-EGFP injection were labeled with antibodies against relevant markers of neuronal subpopulations and glia, and assessed for colocalization with EGFP (Figure 6). EGFP-positive cells were co-localized with β3-tubulin, a general marker for neurons (Figure 6C). Co-localization was not observed with glutamine synthetase, a marker for satellite glial cells (Figure 6R). Thus, our findings indicate that although the VSV-G-pseudotyped vectors have a broad capacity to transduce a variety of cell types *in vitro*, there is preferential transduction of neuronal phenotypes *in vivo*.

EGFP-transduced neurons included co-labeling with markers that identify various neuronal subpopulations. Isolectin B4 (IB4), which recognizes small (23 ± 4 μm diameter in our samples), nonmyelinated non-peptidergic nociceptive neurons (Figure 6I), is bound by 36 ± 16% of transduced neurons expressing EGFP. α-Calcitonin gene-related peptide (CGRP), which recognizes small (23 ± 5 μm), nonmyelinated peptidergic nociceptive neurons (Figure 6L), is expressed by 34 ± 16% of transduced neurons. Transient receptor potential vanilloid 1 (TRPV1), which selectively labels nociceptive neurons (diameter 23 ± 6 μm) that are either myelinated or nonmyelinated (Figure 6O), was detected in 27 ± 8% of transduced neurons. Neurofilament 200 (NF200; Figure 6F), which identifies myelinated neurons, was detected in 40 ± 4% of EGFP-expressing neurons. The transduced population of NF200-positive neurons had diameters (28 ± 12 μm) that were not significantly less than those that were EGFP-negative (32 ± 12 μm; *P *= 0.1), and included large as well as small neurons. In general, the lentivectors showed an ability to transduce a broad population of DRG neuronal phenotypes. Transduction efficiencies within the different subpopulations did not differ (TRPV1: 16 ± 9%; IB4: 25 ± 8%; CGRP: 29 ± 12%; NF200: 17 ± 7%).

#### DRG injection of RABPV-pseudotyped lentivector for in vivo transduction

**Figure 7:**
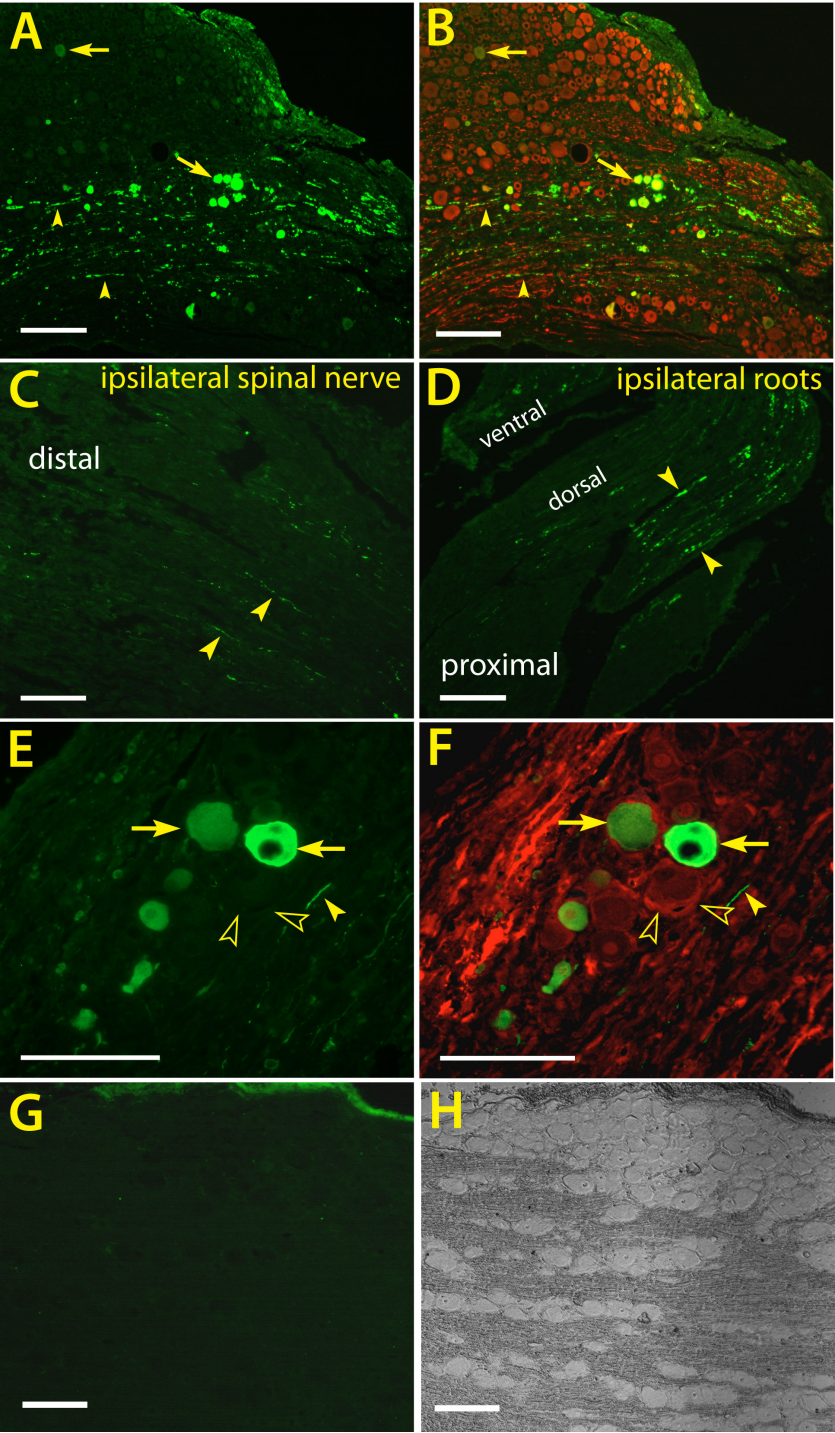
**Direct DRG injection (but not intramuscular injection) of RABPV pseudotyped EF1α lentivector induces EGFP expression in the ipsilateral DRG**. **A, B**. Representative images of EGFP expression in neurons and fibers (**A**, arrows point to neurons and filled arrowheads to fibers) revealed by EGFP IHC and EGFP-expressing neurons and fibers co-labeled with β3-tubulin (**B**, arrows point to neurons and filled arrowheads to fibers) 4 weeks after direct DRG injection of the RABPV coated LV2-EF1α-EGFP. **C, D**. Arrows point to EGFP expression in spinal nerve distal to the injection (**C**) and in nerve roots proximal to the injection (**D**). High magnification shows predominant expression of EGFP in neurons, and no colocalization to glutamine synthetase, a marker for satellite glial cells (**E **and **F**, arrows point to transduced neurons, filled arrowheads to EGFP positive fibers, and empty arrows to glial cells). **G, H**. No EGFP expression was detected in the ipsilateral DRG 4 weeks after gastrocnemius muscle injection of RABPV pseudotyped LV2-EF1α-EGFP, shown in the representative image of EGFP IHC (**G**) and corresponding phase-contrast image (**H**). Scale bars = 200 μm for A, B, = 100 μm for C, D, G, H, and = 50 μm for E and F.

We also studied DRG sensory neuron gene transfer by RABPV pseudotyped LV2-EF1α-EGFP vector directly injected into the DRG. Results showed that RABPV lentivector (2.16 × 10^6 ^TU of purified vector) resulted in similar transduction patterns to those of VSV-G vector, with EGFP expression predominantly in 15 ± 3% (N = 3) DRG neurons and in the nearby nerve roots and spinal nerve (Figure 7).

#### RABPV-pseudotyped lentivector injection into distal muscle for DRG transduction

Lentivectors pseudotyped by Rabies G have demonstrated particular efficacy in transducing spinal cord motor neurons via retrograde transport after peripheral injection into muscle [20]. We therefore examined whether RABPV pseudotyped LV2-EF1α-EGFP might transduce DRG neurons following such injections. Four weeks after gastrocnemius muscle injection of 2.08 × 10^7 ^TU of RABPV EF1α vector, EGFP signal was detectable only in the ipsilateral gastrocnemius fibers (Data not shown), and no EGFP expression was observed in either ipsilateral or contralateral DRGs (Figure 7). This suggests that vector access to peripheral sensory neurons is limited after intramuscular injection of lentivectors, and that intramuscular injection, although effective in delivery to spinal cord motor neurons [20], is not a desirable route for DRG gene transfer by lentivectors.

#### Behavioral evaluation after injection and transduction

**Figure 8:**
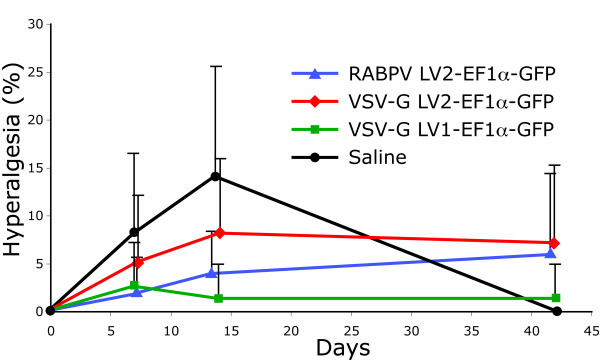
**Pain behavioral evaluation after DRG injections**. The rate of hyperalgesic response to noxious punctate mechanical stimulation transiently increases for animals injected with vehicle (saline + polybrene 100 nM, n = 5), and is minimally affected by VSV-G pseudotyped first-generation lentivector (VSV-G LV1-EF1α-EGFP; n = 10). However, there is a persisting increase after injection of VSV-G pseudotyped second-generation lentivector (VAV-G LV2-EF1α-EGFP; n = 8), or RABPV pseudotyped second-generation lentivector (RABPV LV2-EF1α-EGFP; n = 5). Data are shown as mean ± SD. *P *< 0.05 for ANOVA interaction Group X Time.

Inflammatory changes in the DRG are associated with hyperalgesia in various models of neuropathic pain. Although lentivectors are unlikely to initiate a substantial inflammatory response, we nonetheless examined sensory behavior following injection to identify if viral infection can itself produce manifestation of animal pain. We gauged hyperalgesia by the incidence of a characteristic behavior not seen in normal animals, specifically sustained, complex foot withdrawal with lifting, shaking, or grooming following a noxious punctate mechanical stimulation (a pin touch) to the plantar surface of the hind paw. This method has been validated in neuropathic pain [27], and has been shown to represent an aversive experience and cause conditioned place avoidance [28]. DRG saline injections produced a mild hyperalgesic state (Figure 8) in which up to 14 ± 11% of touches produced a hyperalgesic response, which resolved by 42 days. VSV-G pseudotyped second-generation lentivectors and RABPV pseudotyped second-generation lentivectors produced a mild but sustained increase in hyperalgesia behavior, whereas VSV-G pseudotyped first-generation lentivectors caused no difference in behavior compared to baseline. These patterns were statistically different (repeated measures ANOVA *P *< 0.05 for interaction of injection Group x Time).

## Discussion

Chronic pain, particularly neuropathic pain, has proved resistant to conventional pharmacological approaches. Systemic administration of agents that reverse neuronal hyperactivity frequently produces limiting side effects by their actions on normal neuronal function [29]. Even neuraxial infusions result in widespread distribution in the central nervous system, and also require implantation of complex hardware and ongoing specialized care. Many segmental neuropathic conditions such as radiculopathy, post-herpetic neuralgia, and those related to tumor involvement or trauma, could be addressed by anatomically targeted therapy. By this approach, specific sensory pathways might be subjected to intensive functional modulation while avoiding systemic side effects. The DRG is an appealing therapeutic target. Unlike peripheral nerves or the spinal cord, the DRG is tolerant of needle penetration and injections [18], perhaps in part due to its generous vascular supply [30]. Well-defined clinical injection techniques are available for injecting solutions within or adjacent to the DRG [18]. Furthermore, altered neuronal and glial function within the DRG is a critical component of pain pathogenesis following injury or inflammation of peripheral nerves [31].

Advances in molecular therapies provide new opportunities for highly specific treatment of pain conditions. For instance, therapeutic genes have been introduced into DRGs by administration of viral vectors into the cerebrospinal fluid of the spinal intrathecal space. Although this mode of delivery would be easy to employ clinically, clear limitations include unintended transduction of CNS structures [32,33], low efficiency rates for DRG transduction [34], and uncertain penetration of vectors to the distal pole of the DRG, which could be a particular constraint in clinical settings considering the large diffusion distances in a human DRG. In contrast, direct injection into the DRG has achieved transduction restricted to the DRG and high efficiency in experimental animals [34,35]. Although paresis has also been noted [36], it is not known whether this is vector-related.

Regarding the optimal vector for DRG transduction, it is important to note that the primary types of viral vectors used in previous studies for treating experimental neuropathic pain have been largely episomal in nature, namely adenovirus, herpes simplex virus type 1 (HSV-1) and various serotypes of adeno-associated viruses (AAV). The main drawback in the use of episomal viral vectors, other than the specific vector-related problems, is the potential for limited temporal expression pattern for the transferred gene of interest. For adenoviral vectors, transient vector persistence and transgene expression is largely attributed to the host immune activation due to the presence of early viral gene products in the vector system [37]. However, investigators have designed a helper-dependent or "gutless" adenoviral vectors in which all of the immunogenic viral genes are removed [38-40]. The application of these newer versions of the adenoviral vector to the DRG is now emerging [41]. One of the classic viral vector systems employed to transduce DRG neurons is based on HSV-1 [5,42]. The administration of HSV-1 vectors has typically been through peripheral inoculation, which takes advantage of the natural tropism of HSV-1 for the sensory neurons. Although this vector has shown promise for efficiently transducing these types of neurons, there are specific limitations, which include the difficult and complex nature of manipulating and generating this vector system, vector-related toxicity [43], and potential for conversion of the replication-defective virus into a competent one, depending on the serum status of the infected patients who may have been previously exposed to wild-type HSV.

There is emerging new data investigating the utility of AAV vectors as a therapeutic vehicle to the DRG. These studies document highly efficient transduction of DRG neurons following direct AAV injection into the rat DRG [34,44]. Unlike the other episomal vectors, such as adenovirus and HSV-1, AAV suffers from a restricted packaging capacity (less than 5 kb), potential induction of host immune responses depending on the serotype in humans, and problematic scalability in vector production [4].

To circumvent some of the issues related to the earlier described episomal vector systems, lentivectors have been studied as one of the primary gene therapy vehicles, since its innate ability to integrate into the host genome following cell entry provides the potential for persistent, long-term expression following a single administration [8,45]. Moreover, lentivectors have shown an enhanced propensity to transduce terminally differentiated tissues from neuronal origin [46,47] as demonstrated in the initial discovery by Naldini *et al. *[8], with negligible inflammation [8,20]. The packaging capacity of the lentivector system is relative large (~12-15 kb) [48] and would be suitable for cloning a large majority, if not all, of the currently transferred genes currently undergoing testing in pre-clinical and clinical trials.

In our hands, we successfully transduced DRG neurons using replication-defective lentivectors at ~20% efficiency, which is in contrast with a previous study by Mason *et al. *[35], who estimated much lower levels of transduction (1-2%). The reasons for the higher transduction efficiencies in our study are not clear. Although there are several similarities between the methodologies, such as the same packaging plasmids (pCMVΔR8.74), envelope coat protein, and injection methods in the DRG, a major difference was the time point used to analyze the tissue following lentivector administration. Our study analyzed the DRG tissues at 4 weeks, whereas Mason *et al. *[35] examined their tissues at 2 weeks following lentivector injection. In earlier unpublished experiments in our lab, we observed little to no EGFP expression in the DRG 1-2 weeks after injection, which is consistent with the Mason *et al. *study [35]. It is not clear why EGFP expression following lentivector transduction in the DRG is slow in onset, in contrast to many observations in other mammalian organ systems that demonstrate rapid expression from integrated lentivectors [45,49]. A second explanation for differences between our findings and those by Mason *et al. *[35] could be the difference in the internal promoter used within the lentivector system. We used an EF1α promoter whereas Mason *et al. *used a CMV promoter, which may drive transgene expression in DRG neurons with different strength and temporal activity. Finally, semi-purification of lentiviral particles via OptiPrep ultracentrifugation in our study may also impact the transduction efficiency for *in vivo *applications by removing factors that negatively affect the functionality of the vector [50].

To assess whether a loss of viral accessory genes would be one of the rate-limiting factors delaying the transgene expression by the integrated lentivectors or negatively affect the transduction efficiency to the DRG, we investigated whether including the full complement of viral accessory genes, specifically *vif, vpr, vpu, nef, tat *and *rev*, would have a beneficial role. However, no positive effect on the count of EGFP(+) DRG neurons was calculated following the 4 week injection period by the early-generation lentivectors. The lack of a requirement for viral accessory proteins is consistent with previous *in vivo *studies demonstrating equally efficient transduction using first- or second-generation lentivectors in mouse hepatocytes [17] or rat brain [8]. However, our preliminary behavioral findings suggest that hyperalgesia may be diminished after injection of first-generation vector. This suggests that including the accessory genes may have immune suppressing effects in addition to their role in viral production [17], although the possible added safety of the second generation vector may make this a more desirable choice for translational development.

Although the effectiveness of the lentivector system in our study has equal, or even greater, efficiency than previously described episomal viral systems, it is naïve to believe that the lentivector system does not have its own inherent challenges. First, it is not clear whether insertional mutagenesis will arise following integration, as has been observed in previous clinical trials using simple retroviral vectors based on murine leukemia virus (MLV) [51-53]. However, numerous studies have been published demonstrating that the integration pattern of lentivectors differs from that of the MLV-based vectors [54], so this may not end up becoming a major issue. Other vector systems considered to be largely episomal, including AAV, are also susceptible to the risk of insertional mutagenesis. Specifically, Nakai *et al. *[55] showed that this vector system is also promiscuous in its integration pattern, which may lead to oncogenesis [56]. Second, the role of the host immune system in diminishing lentiviral transduction and/or transgene expression needs further investigation [37]. Finally, since *in vivo *gene transfer into the DRG using lentivectors by our approach only allows for injection of very small volumes, highly concentrated (i.e. several log number higher titer than used in this study) and purified preparations may required for improved gene transfer [57].

In the clinical setting, many clinical neuropathic pain conditions have a segmental presentation, such as nerve trauma, herpes zoster, and tumor involvement, making anatomically focused treatment an appealing option. Standard techniques for delivering agents into the immediate vicinity of the DRG are well established [58], and injection within the DRG is well tolerated [18]. Numerous applications may be envisioned for gene transfer into the DRG. In the area of experimental neuropathic pain research, a myriad of pathogenic triggers and downstream effectors have been proposed [31], often based on imperfect pharmacology in *ex vivo *models. Modulating genetic events at the level of the DRG in behaving animals would provide a new level of anatomic and molecular specificity that may clarify which cellular events are causative in neuropathic pain. In clinical applications, expression in the DRG of secretable analgesic peptides such as neurotrophic factors (e.g. GDNF, VEGF), anti-inflammatory peptides (e.g. IL-4, IL-10, IL-14, fractalkine), inhibitory neurotransmitters (e.g. enkephalin, endomorphin-2), or soluble TNF-α receptor may be most effective, since transduction of all neurons is not a prerequisite for effective treatment. Specifically, the approximately 20% transduction efficiency we demonstrate in our experimental model may be sufficient for providing adequate peptide levels to modulate the performance of all neurons within a ganglion. Alternative approaches may include modulation of intracellular or neuronal membrane peptides through RNAi knockdown of nociceptive-specific targets (e.g. CGRP, NaV1.7, NaV1.8, NF-κB) or overexpression of a naturally analgesic peptide (μ opioid receptor), although the efficacy of this approach would be tightly tied to transduction efficiency.

In conclusion, our study clearly demonstrates that the VSV-G pseudotyped lentivector system can be effectively utilized for genetic modification of sensory neurons *in vivo*. It remains to be explained why the lentivector system expresses the transgene in a delayed manner in comparison to previously published observations in other organs. Nonetheless, our findings suggest that lentivectors delivered to the DRG could be readily employed not only in the study of basic pain mechanisms, but potentially in the treatment of clinical neuropathogenic pain.

## Methods

### Animals

Sprague Dawley rats (5-6 weeks old; 125-150 g body weight) were purchased from Charles River Laboratories (Wilmington, MA). All animal procedures were approved by the Zablocki VA Medical Center Animal Studies Subcommittee and Medical College of Wisconsin IACUC. All rats were allowed *ad libitum *access to food and water prior to and throughout the experimental protocol.

### Lentivector production and purification

#### Lentivector transfer plasmids

The lentiviral transfer vector plasmids used in this study expressed the enhanced green fluorescent protein (EGFP) and was modified to include *cis*-acting DNA elements that would promote increased transduction efficiency and transgene expression. Each of the transfer plasmids contained central polypurine tract sequences 5' to the promoter and woodchuck post-regulatory elements 3' to the transgene. In addition, the transfer plasmids had 3' LTR deletion to minimize promoter activity and lead to self-inactivation following integration. These changes to the transfer plasmids are described in the references for each construct: 1) pEF1α-EGFP (Addgene plasmid 12255) has the elongation factor 1a promoter; 2) p156CAG-EGFP (provided by Dr. Inder Verma, Salk Institute) was described previously [59] and contains the composite hybrid chicken β-actin promoter fused with CMV enhancer elements; 3) pHR(+).c.UbC.EGFP.R(-)W(+) contains the human Ubiquitin C promoter [60]; and 4) pHR(+).c.mPGK.EGFP.R(-)W(+) contains the mouse phosphoglycerate kinase 1 (PGK) promoter and was cloned using standard cloning techniques.

#### Packaging and envelope plasmids

The packaging plasmids used in this study were either first-generation (pCMVΔR8.2) [8] or second-generation (pCMVΔR8.74) [61]. The envelope plasmids are as follows: 1) pVSV-G contains the vesicular stomatitis virus G protein; 2) pLCMV-GP contains the lymphochoriomeningitis virus envelope [62]; 3) pCEF-rabies PV contains the glycoprotein from rabies virus PV strain (RABPV) [63]; and 4) pCEF-rabies SAD (B19) contains the rabies SAD glycoprotein (RABSAD) [64].

#### Lentivector production, purification and titering

Briefly, human embryonic kidney 293T cells (ATCC) were triple-plasmid transfected into 100 mm dishes using the calcium phosphate method previously described by us [17,60,65] using chloroquine (25 μM). The three plasmids transfection protocol consisted of a combination of a transfer plasmid (10 μg), packaging plasmid (6.5 μg) and envelope plasmid (3.5 μg) per dish. The media was replaced 12-16 hours after the initial transfection, and then 24-48 hours later, the supernatants were collected, pooled when applicable, and cleared by slow speed centrifugation at 1,500 rpm for 5 minutes. Subsequently, the conditioned media was filtered through a 0.45-μm pore, and either frozen in aliquots at -80°C or if concentration was necessary, the filtered conditioned media was centrifuged at 23,000 rpm for 2 h in a swinging bucket rotor (SW28 Beckman, Fullerton, CA). The final pellet was resuspended in Ca^2+^/Mg^2+ ^free phosphate buffered saline (PBS). For the *in vitro *primary DRG dissociated cell cultures and *in vivo *injections, the lentiviral particles were further purified by a modified OptiPrep (Sigma-Aldrich, St. Louis, MO) density gradient ultracentrifugation [50]. OptiPrep was used due to its demonstrated safety in human clinical trials [66]. Briefly, the concentrated lentivector preparations in 5 ml PBS were mixed with equal volume of 60% OptiPrep to yield a 30% OptiPrep solution, and then 2 ml of 5% OptiPrep (in PBS) was laid on top. The gradient was spun at 37,000 rpm in a Beckman ultracentrifuge using a SW 41Ti rotor for 2 h at 4°C. Lentiviral particles formed a buoyant dense band at 5%/30% interface and were collected. The collected fraction was applied to a Centricon (100-cutoff) filter, centrifuged for 10 min at 10,000 × *g *until ~100 μl remained. The final product was stored in aliquots at -80°C or used immediately for injections. Functional titers were determined by limiting dilution on HeLa cell (2 × 10^5 ^cells/well) in the presence of polybrene (8 μg/ml). The transduced HeLa cells were compared to a reference naïve HeLa cell line and analyzed by fluorescence-activated cell sorting (FACS) analysis. The titers were calculated as transduction units (TU) per ml. Each of the lentivector batches utilized in our experiments had comparable titers ranging from 7.6 × 10^5 ^to 1.6 x10^6 ^TU/ml (conditioned media) for *in vitro *experiments using 50B11 cells and from 3.43 × 10^8 ^to 2.65 × 10^9 ^TU/ml (purified concentrated vectors) for *in vitro *primary DRG dissociated cell cultures *and in vivo *injections. The lentivector suspensions were briefly centrifuged and kept on ice immediately before exposing to cells and injection to the rats.

### Transduction of cultured DRG neurons

#### Immortalized dorsal root ganglion sensory neuronal cell (50B11) cultures

50B11 cells are immortalized DRG neuronal lines from embryonic day 14.5 rats, and were kindly provided by Dr. Ahmet Höke (Department of Neurology, Johns Hopkins University). The 50B11 cells have been reported to phenotypically exhibit characteristics similar to that of nociceptive neurons [21,22]. 50B11 cells were grown to 70% confluence and maintained in DMEM/F12 medium (Invitrogen, Carlsbad, CA) supplemented with 10% fetal bovine serum (FBS), 100 U/ml penicillin-100 μg/ml streptomycin, 0.2% glucose, 0.5 mM L-Glutamine, 1X B-27 supplement (Invitrogen). Neuronal differentiation of 50B11 cells were induced by addition of 50 μM of forskolin (Sigma-Aldrich) in the culture medium [21]. Cell monolayers were fixed in 10 min with 4% paraformaldehyde, permeablized for 1 h with 0.5% Triton X-100, and then stained for DRG neuronal markers by a standard immunofluorescence protocol as described below using antibodies against CGRP, TRPV1, and β3-tubulin, or isolectin IB4 binding. For lentiviral transduction, cells were plated at a density of 1 × 10^5 ^cells per well onto 24-well plates for 24 h. Cultures were serially transduced over a two day period (one transduction per day) with conditioned media containing the various modified lentivector systems at a MOI of 1 at each infection in the presence of polybrene (8 μg/ml of culture medium). After four days *in vitro*, cells were collected, and the transduction efficiency by the lentivectors in the 50B11 cells was analyzed by FACS.

#### Dissociated DRG cell culture and lentivirus transduction

Cultures of rat DRG neurons were prepared as described previously [67] with some minor modifications. In brief, DRGs from the lumbar segments of male Sprague Dawley rats were obtained under sterile conditions. DRGs were cut into approximately 2 mm sections, and digested in a collagenase mixture (0.5 mg/ml; Liberase Blendzyme 2; Roche, Indianapolis, IN) dissolved in serum-free DMEM at 37°C for 30 min. Digested ganglia were collected by centrifugation at 1,000 × *g *for 5 min and dissociated in 0.25% trypsin mixed with serum-free DMEM for 30 minutes under the same conditions described earlier. At the end of the 30 minutes, the trypsin was inactivated by adding soybean trypsin inhibitor (5 mg/ml). The dissociated-cell suspension was centrifuged at 1,000 × *g *for 5 min, and the resulting pellet was resuspended in pre-warmed serum-free growth medium (Neurobasal A; Invitrogen, Carlsbad, CA) supplemented with B27, 100 U/ml penicillin-100 μg/ml streptomycin, 5 mM glutamine and 30 ng/ml NGF-β). A single cell suspension was achieved by gentle titration and plating onto poly-L-lysine coated glass coverslips. Cultures were transduced with OptiPrep-purified and various modified lentivectors in the presence of polybrene (4 μg/ml of culture medium) 10 h after cultures were established. Eighteen hours after the onset of transduction, the viruses were removed and were replaced with supplemented neurobasal medium. After five days *in vitro*, direct EGFP fluorescence was examined and photographed under an inverted Nikon TE2000-S epifluorescence microscope attached to an Imaging camera MicroFire Picture Frame imaging software (Optronics Microfire, Santa Barbara, CA). Images were analyzed and processed in Adobe Photoshop CS3.

To accurately determine the transduction efficiency of various lentivectors on neurons or glial cells in dissociated adult rat DRG cultures, four independent vector-treated cultures were stained using either neuron-specific β3-tubulin monoclonal antibody or rabbit anti-glutamine synthetase (GS) antibody, a ubiquitous satellite glial cell marker. In order to derive the percentage of transduced neurons, the total number of EGFP-positive neuronal cells visible in 5 randomly fields (10×) *per *culture well was counted, cells showing colocalization with β3-tubulin were determined using color overlay on individual images, and the mean percentages of EGFP/β3-tubulin positive neuronal cells were calculated. For counting glial cells following GS immunostaining, the fluorescent dye Hoechst 33342 (0.25 μg/ml, Invitrogen) was used for nuclear staining to correctly distinguish the glial cells based on their smaller nuclear bodies and high-density staining by Hoechst from the other cells (neurons and fibroblasts with larger but lightly stained nuclei) in the culture wells, EGFP positive glial cells colocalized with GS in the merged pictures were counted in 5 randomly fields (20×) per culture well, and results represented by average percentage of colocalization with GS.

### Injection of lentivectors *in vivo*

The DRG microinjection of lentivectors was performed as previously described [19]. In brief, male Sprague Dawley rats were anesthetized under isoflurane, and subsequently a lower lumbar incision was made a few millimeters to the right of the midline. The paraspinal muscles were separated to expose the lateral aspect of the L4 and L5 vertebrae and their transverse processes, exposing the L4-L5 intervertebral foramen for injections of the L5 DRGs. After exposure, the intervertebral foramen was enlarged by removal of laminar bone, exposing the distal half of the ganglion. A micropipette was advanced approximately 100 μm into the ganglion and held in position for 3-5 min. Injections contained either lentivectors (2 μl) or saline (2 μl), in both cases containing polybrene (100 ng), which has been used as a standard reagent to improve *in vivo *transduction with lentivectors [68-70]. Injection was performed over a 5 min period using a Nanoliter 2000 microprocessor-controlled injector (World Precision Instruments, Sarasota, FL). The pipette remained in place for 5 min to allow the pressure within the ganglion to equalize, and then the pipette was slowly removed. Muscles and fascia were closed using 4-0 chromic gut suture and the skin was closed with staples. Unilaterally left gastrocnemius muscle injection was performed in rats using a microsyringe fitted with a 30-gauge needle (Hamilton) [20], inserted through the skin. Injected solution consisted of 20 μl of RABPV pseudotyped EF1α-EGFP lentivector containing a total of 2.08 × 10^7 ^viral particles (TU) over 90s.

### Characterization of EGFP expression

#### Immunohistochemistry (IHC) and Imaging

Four weeks after injection, animals were terminally anesthetized and transcardially perfused with 4% paraformaldehyde (PFA) in PBS. DRGs and spinal cord segments, as well as gastrocnemius muscle (RABPV EF1α-EGFP injection) were dissected, post-fixed in 4% PFA, and processed for paraffin embedding and sectioning. Detection of EGFP in tissue sections was performed by EGFP immunohistology in order to achieve an improved signal-to-noise resolution compared to direct detection of EGFP fluorescence. The EGFP signal on DRG sections was detected using a primary mouse anti-EGFP antibody (1:400, Santa Cruz Biotechnology (SCB), Santa Cruz, CA), and subsequent incubation with Alexa Fluor 488-conjugated donkey anti-mouse second antibody (1:2,000, Jackson ImmunoResearch, West Grove, PA). IHC enhanced EGFP signals were co-labeled using the following antibodies: mouse anti-β3-tubulin (1:400, SCB), mouse anti-α-calcitonin gene-related peptide (CGRP, 1:100, SCB), rabbit anti-vanilloid receptor subtype 1 (TRPV1, 1:200, Thermo-Fisher scientific, Pittsburgh, PA), mouse anti-neurofilament 200 (NF200, 1:1,000, Sigma-Aldrich), and rabbit anti-glutamine synthetase (GS, 1:600, SCB). For co-localization, primary antibodies were revealed by the appropriate 549-conjugated secondary antibodies (1:2,000, Jackson ImmunoResearch). Non-peptidergic neurons were labeled using biotinylated griffonia simplicifolia Isolectin B4 (IB4) (10 μg/ml, Invitrogen) coupled to Alexa Fluor 549-conjugated Streptavidin (1:6,000; Jackson ImmunoResearch). Standard fluorescent immunohistochemistry techniques were used for all protocols as described previously [71,72], with BSA replacement of first antibody as the negative control. Briefly, paraffin sections (5 μm thick) were deparaffinized in xylene, rehydrated through graded alcohol, and processed for antigen retrieval by heating the sections at 95°C in 10 mM sodium citrate (pH 6.0) for an initial 5 min and for two successive 5 min periods. Endogenous autofluorescence was suppressed by dipping of slides in 2% picric acid for 10 min. After washing, sections were blocked with 3% BSA for 1 h at room temperature, and then incubated overnight at 4°C with primary antibody or biotinylated IB4 diluted in 3% BSA. Sections were then washed in PBS, incubated for 1 h at room temperature (RT) with corresponding 549-conjugated secondary antibody, or 549-conjugated streptavidin, diluted in 1% BSA, and then dehydrated through an ethanol series to xylene and mounted. The sections were examined and images captured under a Nikon TE2000-S fluorescence microscope with filters suitable for selectively detecting the fluorescence of 488 (green) and 549 (red) or were examined under a light microscope. For co-localization, images from the same section but showing different antigen signals were overlaid.

#### Immunoblotting of EGFP protein in transduced DRG samples

DRG tissue was harvested following transduction with VSV-G lentivectors carrying the EF1α-EGFP cassette after 4-week following injection. Protein lysates were extracted using 1X RIPA buffer (150 mm NaCl, 2 mm EDTA, 1% Nonidet P-40, 1% sodium deoxycholate, 0.1% SDS, 50 mm NaF, 10 μg/ml aprotinin, 10 μg/ml leupeptin, 1 mm phenylmethylsulfonyl fluoride, and 20 mm Tris-HCl buffer, pH 7.4). As a positive control for EGFP expression, HeLa cells transduced with the same lentivector was extracted at the same time. Protein concentration determined by using the BCA kit (Pierce, Rockford, IL). DRG and HeLa protein lysates (20 μg) were size separated using a 4-12% gradient SDS-PAGE gel, transferred to 0.22 μm nitrocellulose membrane, and blocked in 5% skim milk. The blots were subsequently incubated overnight at 4°C with a polyclonal rabbit anti-GFP antibody (1:1,000; Cell Signaling, Danvers, MA) or mouse monoclonal anti-α-tubulin antibody (1:1,000; SCB). Immunoreactive proteins were detected by enhanced chemiluminescence (Pierce, Rockford, IL) after incubation with either HRP-conjugated anti-rabbit IgG (1:2000, SCB) or anti-mouse IgG (1:5,000; SCB).

#### Histological quantification

L5 DRGs from three to four animals were analyzed for quantification. To evaluate transduction rates, every fifth DRG section was selected from the consecutive serial sections (7-10 sections for each DRG), and in each selected section, the number of EGFP fluorescent cells was counted and transduction efficiency was expressed as the percentage of EGFP immunopositive cells in the total neuronal profiles revealed by β3-tubulin [33,73]. Every section was photographed at fixed exposure settings of 10× magnification in which most of neurons (100~300 neuron profiles) in each section were covered, by use of a Nikon TE2000-S epifluorescence microscope. When counting, image contrast was adjusted (Adobe Photoshop CS3) such that background levels became inapparent, and the same cutoff level was used for all images [74,75]. Rates for EGFP expression in neuronal subpopulations were determined in a similar fashion using specific antibodies in at least 3 sections. All counting was done using a masked protocol and the average from two observers was used for calculation. Diameters were derived from the neuronal area ($=2\sqrt{\left(area∕\pi \right)}$) measured only in profiles for which a nucleus was evident.

### Behavioral analysis

Noxious punctate mechanical stimulation was performed using the point of a 22 g spinal anesthesia needle, which was applied to the center of the paw with enough force to indent the skin but not puncture it. This was applied for 5 applications separated by at least 10s, which was repeated after 2 min, making a total of 10 touches [27]. For each application, the induced behavior was either a very brisk simple withdrawal with immediate return of the foot to the cage floor, or a sustained elevation with grooming that included licking and chewing, and possibly shaking, which lasted at least 1s, characteristic of hyperalgesic behavior [27]. The degree of hyperalgesia was recorded as the percentage of total touches that were of this second complex and sustained type, which is also associated with aversion in a conditioned place avoidance paradigm [28].

### Data analysis

Data are expressed as means ± SD. The statistical significance of differences for the transduction efficiencies on 50B11 cells and primary DRG cell cultures by different modifications was assessed by ANOVA, and significance of *in vivo *transduction rates between LV1-EF1α-EGFP and LV-EF1α-EGFP were analyzed by the unpaired Student's t-test, using Statistica (StatSoft, Tulsa, OK). Behavioral changes over time in each group were analyzed by repeated measures ANOVA.

## Competing interests

The authors declare that they have no competing interests.

## Authors' contributions

QH, FP and HY conceived of the study and designed the experiments. HY, GF, GJ, FP, and QH carried out the experiments. JR provided plasmids to generate the alternative pseudotyped lentivectors and provided expertise in their production. GF and QH performed statistical analyses. FP, QH, and HY prepared the manuscript. QH and FP obtained grant support for this study. All authors read and approved the final manuscript.

## Supplementary Material

Additional file 1 **Characterization and lentivector transduction of immortalized DRG neuronal cells (50B11 cells)**. Phase images of undifferentiated (**A**) and differentiated (**B**) 50B11 cells show neuronal-like morphology with extension of axons after differentiation with forskolin. Immunofluorescence images exhibit 50B11 cells stained with IB4 (**C**, red) and TRPV1 (**D**, red) in undifferentiation, and 3-tubulin (**E**, green) and CGRP (**F**, green) after differentiation. EGFP expression images of 50B11 cells 72 h after lentivector transduction (MOI = 2) show the relative transduction activity of VSV-G pseudotyped lentivectors incorporating various cellular promoters including EF1 (**G**), UbC (**H**), PGK (**I**), and CAG (**J**), or lentivectors containing same EF1 promoter but pseudotyped with different envelope glycoproteins including VSV-G (**K**), RABSAD (**L**), RABPV (**M**), and LCMV (**N**). Scale bars = 100 μm. Click here for file
