# Supplementary figures and images for: Lentiviral gene transfer into the dorsal root ganglion of adult rats
Source: Mol Pain. 2011 Aug 23;7:63. doi: 10.1186/1744-8069-7-63 (PMC3179738; doi:10.1186/1744-8069-7-63)

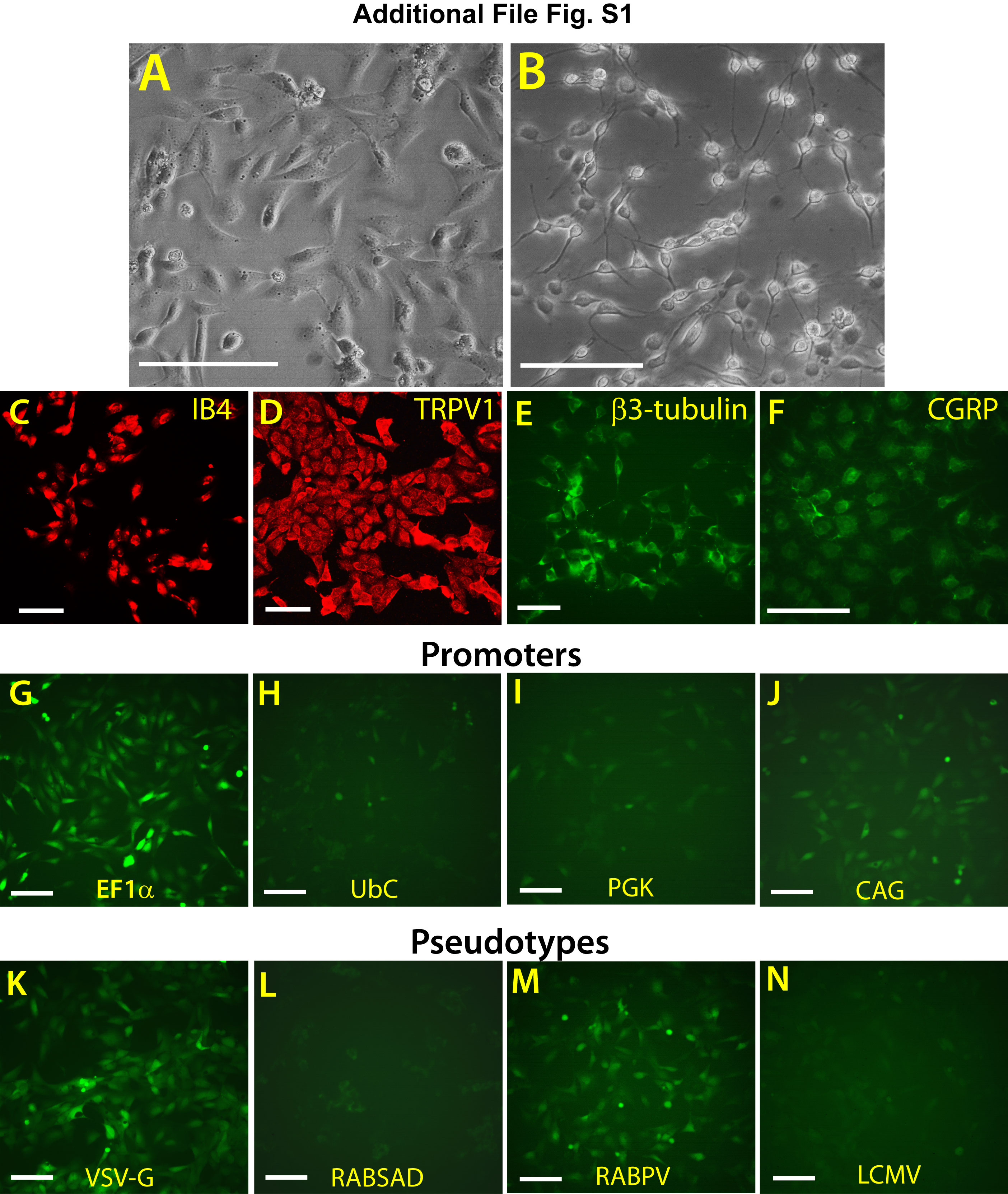

Supplement: Additional file 1 — Characterization and lentivector transduction of immortalized DRG neuronal cells (50B11 cells). Phase images of undifferentiated (A) and differentiated (B) 50B11 cells show neuronal-like morphology with extension of axons after differentiation with forskolin. Immunofluorescence images exhibit 50B11 cells stained with IB4 (C, red) and TRPV1 (D, red) in undifferentiation, and 3-tubulin (E, green) and CGRP (F, green) after differentiation. EGFP expression images of 50B11 cells 72 h after lentivector transduction (MOI = 2) show the relative transduction activity of VSV-G pseudotyped lentivectors incorporating various cellular promoters including EF1 (G), UbC (H), PGK (I), and CAG (J), or lentivectors containing same EF1 promoter but pseudotyped with different envelope glycoproteins including VSV-G (K), RABSAD (L), RABPV (M), and LCMV (N). Scale bars = 100 μm. [file 1744-8069-7-63-S1.JPEG]
